# Sodium Intake and Related Diseases 2.0

**DOI:** 10.3390/ijms23010170

**Published:** 2021-12-24

**Authors:** Alessandra Durazzo, Ginevra Lombardi-Boccia, Antonello Santini, Massimo Lucarini

**Affiliations:** 1 CREA–Research Centre for Food and Nutrition, Via Ardeatina 546, 00178 Rome, Italy; 2 Department of Pharmacy, University of Napoli Federico II, Via D. Montesano 49, 80131 Napoli, Italy

Many statements have been reported in literature from various sources warning of the possible risk to health connected to high salt (as sodium chloride) intake in the everyday diet, and it is increasingly pressing [1]. In general, on a worldwide basis, most of the adult population consumes between 8 and 15 g of salt per day, when the WHO recommends a value of less than 5 g per day [2].

It is well known that high dietary sodium can induce a rise in blood pressure (BP) but, independent of a change in BP it may also cause target organ damage, with direct effects on the brain, heart, kidneys, vasculature, skin, and bone, contributing to disease development over time [3,4].

The mechanisms underlying sodium-induced health consequences are not yet completely understood, but they could involve alterations in organs’ function through common mediators of the target organ dysfunction, including heightened inflammation and oxidative stress.

Hyseni et al. [5], in a systematic review on dietary salt reduction policies, remarked on how comprehensive strategies involving multiple components (reformulation, food labeling, and media campaigns) and “upstream” population-wide policies, e.g., mandatory reformulation, generally appear to achieve larger reductions in population-wide salt consumption than “downstream”, individually focused interventions.

It is worth mentioning a paper tackling salt consumption outside the home [6]. Ali et al. [7] reviewed the application of mobile health technologies aimed at salt reduction.

Among recent studies, the paper of Dodd et al. [8], published in *Advances in Nutrition*, presents a systematic review of the evident effectiveness and feasibility of taxing salt and foods high in sodium. Modeling suggests that taxing all foods based on their salt content is likely to have more impact than taxing specific products high in salt, given that salt is pervasive in the food chain. Further research is needed in this direction [8].

Donfrancesco et al. [9] reported the trend of salt intake measured by a 24-h urine collection in the Italian adult population between 2008 and 2018 in the framework of the CUORE project surveys. The average daily salt intake of the general Italian adult population remains higher than the WHO recommended level, but a significant reduction of 12% in men and 13% in women has occurred over the past ten years.

A current cluster-randomized controlled trial study protocol carried out in a rural area of Bangladesh by Islam et al. [10] reported a lowering of blood pressure through changing lifestyle by means of a motivational education program.

The recent review of Zhang et al. [11] studied and reported how excessive consumption of high-sodium foods during COVID-19 presents an underappreciated public health risk.

This second edition of the proposed Special Issue is specifically aimed at gathering and assessing recent research on the molecular-level mechanisms of the relationship between sodium intake and related diseases; e.g., studies of the role of sodium in the physiological processes, with a focus on the mechanisms of action, are welcome.
